# Metabolic costs of spontaneous swimming in *Sprattus sprattus* L., at different water temperatures

**DOI:** 10.1371/journal.pone.0225568

**Published:** 2019-11-22

**Authors:** Laura Meskendahl, René Pascal Fontes, Jens-Peter Herrmann, Axel Temming

**Affiliations:** 1 Institute for Marine Ecosystem- and Fisheries Science, University of Hamburg, Olbersweg, Hamburg, Germany; 2 Reederei Laeisz GmbH, Rostock, Germany; Penn State University, UNITED STATES

## Abstract

Oxygen uptake (*M*O_*2*_; mgO_2_ fish^-1^h^-1^) of fish groups was measured at temperatures between 10–19°C in an intermittent-flow respirometer to quantify the metabolic costs of spontaneous swimming patterns in the small clupeid *Sprattus sprattus*. Movements of individual fish within the school were tracked automatically during respirometry. Oxygen uptake was then related to mean swimming speeds and the number of sharp turns (>90°), which are common behavioural elements of spontaneous swimming in clupeid fish. Different possible model formulations for describing the relationship between respiration and swimming patterns were compared via the AIC. The final model revealed that costs for sharp turns at a frequency of 1 s^-1^ doubled the metabolic costs compared to those with zero turns but with likewise a moderate swimming speed of 0.28 body length ^-1^. The cost for swimming doubled if the swimming speed was doubled from 0.28 to 0.56 BLs^-1^ but increased by a factor of 4.5 if tripled to 0.84 BLs^-1^. Costs for transport were minimal at a speed of 0.4 body lengths s^-1^ at all temperatures. New basic input parameters to estimate energy losses during spontaneous movements, which occur typically during foraging in this small pelagic fish, are provided.

## Introduction

Fish swimming costs in bioenergetic models are often estimated from empirical relationships between oxygen uptake (*M*O_2_) and forced swimming speeds. Alternatively, simple activity multipliers have been applied to account for the metabolic costs of swimming above maintenance costs [1–3] when no species-specific information was available. Activity costs during spontaneous movements, however, may be larger and more variable than those of steady swimming [4–6] and variables other than average swimming speed can be important descriptors for estimating *M*O_2_ during spontaneous activity. For small pelagics, such as *Sprattus sprattus* (Linnaeus, 1758) or juvenile *Clupea harengus* (Linnaeus, 1758), spontaneous movements represent common behaviours during active feeding [7,8], searching for food or escaping from predators [9]. Planktivorous fishes may in fact spend most of their time feeding continuously on single small particles in contrast to piscivorous fish, which consume large items less frequently. Hence, unsteady swimming with low to moderate speeds and frequent changes in speed and direction [6,9,10] may be the dominant behaviour in planktivorous fishes. During unsteady swimming, active metabolic rate (AMR) will be reached at relatively low swimming speeds compared to steady swimming as spontaneous activity includes expensive manoeuvres in terms of metabolic costs, such as changes in speed and direction [11–13]. Manoeuvring during foraging is therefore more costly than swimming at a fixed velocity is [14]. For *S*. *sprattus*, being the dominant small pelagic fish in the Baltic Sea [15], metabolic costs for spontaneous swimming patterns, such as frequent turns, have not yet been determined and were not included in bioenergetic models. The predation impact of *S*. *sprattus* was so far estimated from stomach contents and gastric evacuation models [16] or taken from bioenergetics models with parameters largely borrowed from other species [17].

Technically, it is very difficult to deduce the activity cost of zooplanktivorous fish from oxygen uptake rates measured directly during feeding because typical feeding behaviour requires a large water volume. This constrains longer measuring intervals within the respirometer and the measurements of activity costs are confounded with the uncontrolled respiration of live prey organisms, bacterial respiration of dead prey items and the effects of SDA. We therefore developed a different approach by firstly estimating the costs for spontaneous swimming patterns (current study) of unfed fish. In a second step, the occurrences of different swimming patterns during feeding were determined in separate experiments in a larger water volume [8]. The advantage of this approach is that the determination of feeding associated swimming patterns did not require respirometry, which would limit the size of the experimental tank in a way that natural feeding behaviour would be impossible to observe. Previous investigations revealed that oxygen uptake rates of fish correlate with various movement patterns like turning rates [18], tail beat frequency [19–21], pectoral fin beat frequency [22], tail beat angle [12] or tail beat pressure [23]. Costs for such movement patterns were so far not quantified for schooling fish in detail.

The objective of the present study was therefore to relate oxygen uptake of *S*. *sprattus* to spontaneous swimming patterns utilizing video observations and respiration rates from the start phase of experiments.

## Materials and methods

### Ethic statement

The experiment was conducted in 2005, well before the more rigid law for animal protection was put in force in 2013. Under the previous legislation from 1972 our type of experiment was not regulated since it did not involve treatments or interventions that cause pain, suffering or harm to the animals. The new regulation introduced in 2013 is much more rigid and today we need explicit permits for both keeping the fish and for conducting such low level experiments. We have conducted two different experiments after 2013 with very similar experimental treatment of the fish investigating growth and feeding behaviour with the same species, and for both we have received explicit permits (numbers 58/14 and 59/14, Amt für Verbraucherschutz, Freie und Hansestadt Hamburg)

### Fish capture and maintenance

Young of the Year (YoY) juvenile *S*. *sprattus* were caught in the Kiel Bay (Baltic Sea, 54° 29’N; 10° 15’E) by a hand operated dip net with a mesh size of 6 mm. Fish were then transferred within a 700 L circular tank with aerated seawater to the aquarium facility at the University of Hamburg, Institute of Hydrobiology and Fisheries Science. *S*. *sprattus* were held in large groups in circular tanks (diameter 1.5 m) connected to the recirculating system of the institute at a salinity of 16 psu and a temperature of 16°C more than twelve months prior to the start of experiments. Fish were fed twice a day with pellet food (LARVIVA, DANA FEED A/S) and once a day with live freshly hatched brine shrimp nauplii (*Artemia* sp.; INVE Aquaculture). The light rhythm was fixed to 13 hours light and 11 hours dark.

### Respirometry and experimental design

**Fig 1:**
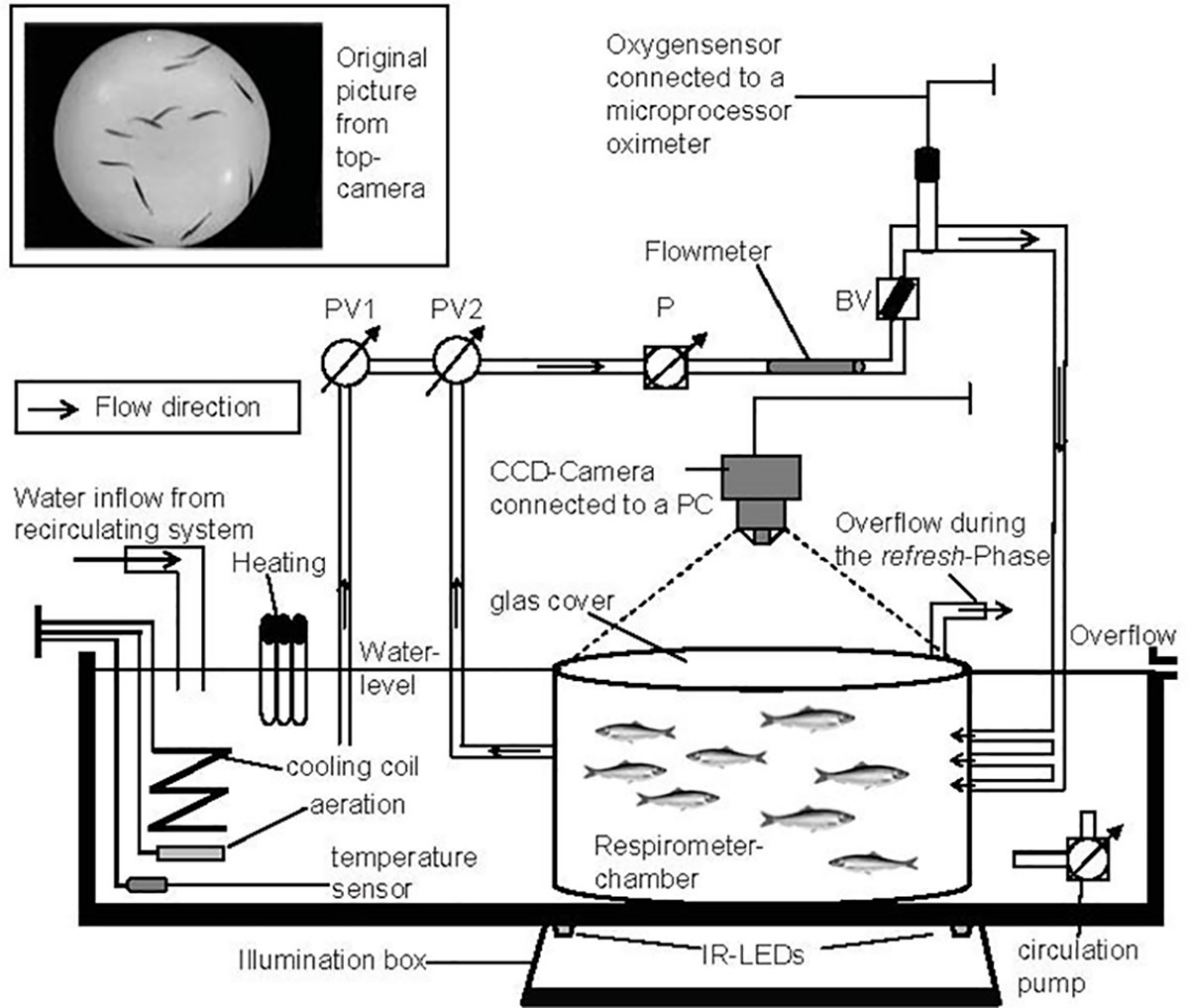
Schematic of the intermittent-flow respirometer. Images were captured with a frame rate of 4 Hz by use of a CCD Camera connected to a PC. Illumination for the Camera was given by infrared-LEDs under the respirometer chamber. This allowed a bright and constant background for which fish appeared as dark objects. PV1 and PV2 are computer controlled pneumatic valves. During the measuring phases (23 min) PV1 was closed and opened during refresh-Phases (5 min), so that oxygen-saturated water from the surrounding tank was pumped into the respirometer chamber. P = pump, BV = ball valve.

**Fig 2:**
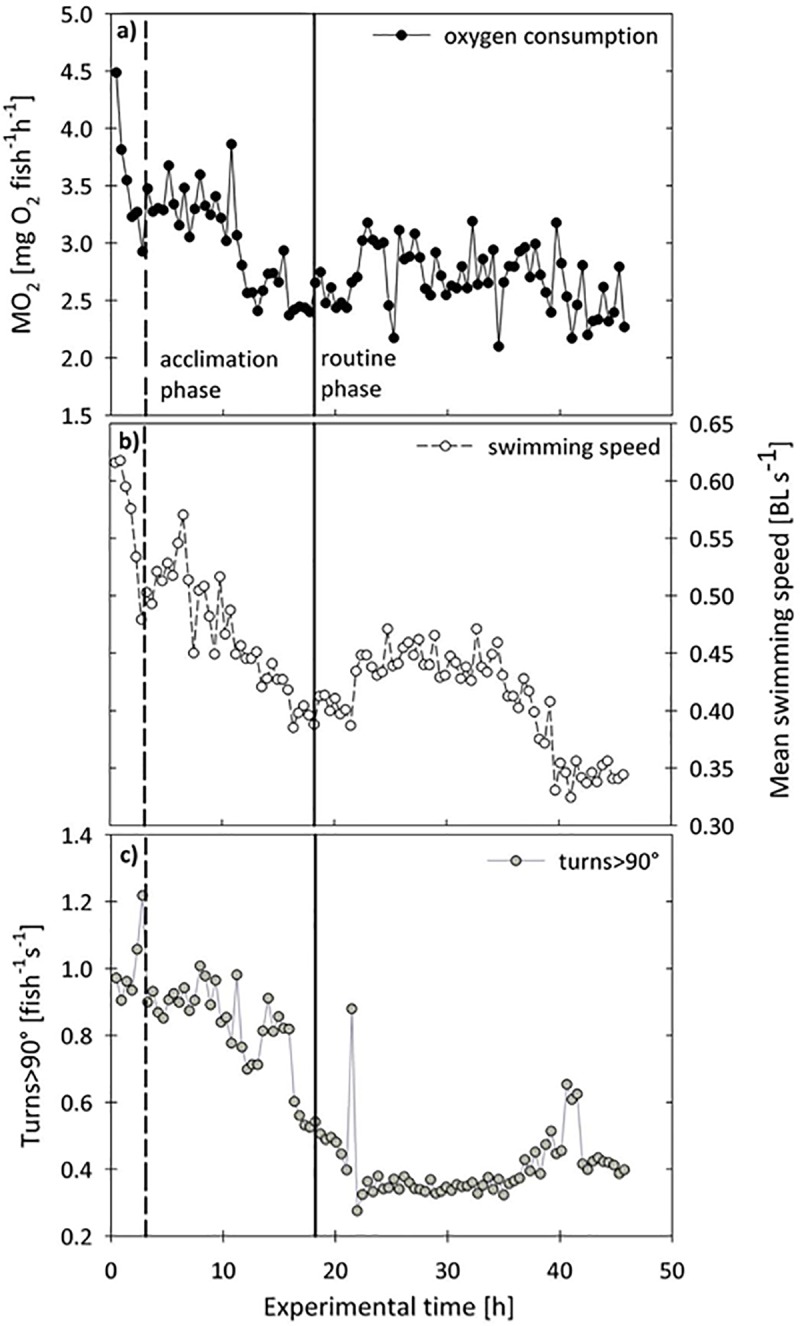
Oxygen uptake of group of sprat at 19°C. (**a)** *M*O_2_ (mgO_2_ fish^-1^ h^-1^) over experimental time (h), (**b)** the corresponding mean swimming speeds (body length s^-1)^ and (**c**) turns >90°(fish^-1^s^-1^). The vertical solid line indicates the beginning of the routine phase used for SMR calculations (Meskendahl 2010); the dashed line separates the initial period including possible effects of handling stress on *M*O_2_. For analysis of activity related metabolic rates of sprat only values from the acclimation phase after exclusion of the first 2–6 measurements (initial period) were used. The separation procedure between routine and acclimation phase followed the description in Herrmann and Enders (2000).

Fish were acclimatized to experimental temperature (10, 13, 16 or 19°C) and smaller tank size (80–100 l) for two weeks and were not fed 48 h prior to experimentation in order to avoid post feeding effects. *S*. *sprattus* is a schooling fish, so that testing in groups was necessary as unfamiliarity can increase *M*O_2_ [24]. The influence of temperature and activity on metabolic rates of *S*. *sprattus* was quantified by measuring oxygen uptake of a group of 8–13 fish in an intermittent-flow-respirometer [25–27] with a chamber volume including all pipes of 99.9 litres. The circular respirometer chamber had a radius of 27 cm and a height of 39 cm and was submerged in a larger tank, connected to the seawater circulating system (Fig 1). The chamber dimensions allowed measuring a significant decrease in oxygen uptake of a small group of fishes over a preferably short measuring time. A flowmeter (B.I.O.-Tech, VISION2000) was inserted between the pump and the oxygen sensor in order to adjust the water flow rates through the chamber, which indirectly controlled the water velocity. All measurements, including the flowmeter count (l min^-1^), were recorded on a PC with the measuring software ARGUS (SORCUS Computer GmbH, Version 4.0), which also controlled all devices and monitored all variables. Each experiment lasted for at least 45 hours with alternating refreshing (5min) and measuring intervals (23 min). The length of each measuring interval was set to 23 min in order to have a significant decrease in oxygen concentration, but also the highest possible data resolution. During refreshing intervals oxygen-saturated water from the surrounding tank was pumped into the respirometer chamber to re-establish the oxygen saturation to 95–100%. After a short delay (3 min) allowing for homogenous mixing in the chamber, the oxygen decline was interpreted as respiration rate. Within each measuring period, the oxygen saturation and the water temperature were measured by a microprocessor oximeter (WTW, Oxi 539) and recorded with a frequency of 1 Hz. Experiments were performed at temperatures of 10, 13, 16 and 19°C and were divided into acclimation periods and routine phases (Fig 2) based on procedures described by Herrmann & Enders [28]. Results of the data collected during the routine phase of experiments (Fig 2) were previously published [27] and are not presented here. During the routine phases fish activity was relatively constant, so that no clear trends were detectable between respiration and activity. In contrast, during the acclimation period, decreasing oxygen uptake rates were associated with decreasing swimming speeds and turning rates (Fig 2). This corroborated the assumption that the elevated metabolism during this period was mainly caused by elevated swimming activities of the fish. Only values from the first 7–28 hours of each experiment were used for further analysis depending on the beginning of the routine phase (and less variable measurements) and the behaviour of fish.

As *S*. *sprattus* is a highly sensitive fish susceptible to scale loss, it was not possible to select individuals of exactly the same body mass. Thus, fish groups of the different experiments had mean wet masses ranging from 6.90 to 9.76 g (Table 1) and an overall mean total length of 10.6 cm (± 0.3). After each experiment fish were removed and rapidly killed by an overdose of anaesthetic (MS-222 at >0.2 g l^-1^). The respirometer was closed again without cleaning and oxygen uptake was measured for at least 24 h to determine bacterial respiration. For each temperature one value for bacterial respiration was calculated as average value and subtracted from the total oxygen uptake rate of the preceding experiments [27].

**Table 1:** Metabolic rates per experiment.

Exp	*n*	Mean	T	*M*O_2_	*M*O_2_	Range of *U*		Range of *M*	
No		M_W_ ± SD	[°C]	± SD		Min	Max	Min	Max
		[g]		[mgO_2_ fish^-1^h^-1^]	[mgO_2_ g^-1^h^-1^]	[BL s^-1^]	[BL s^-1^]	[fish^-1^s^-1^]	[fish^-1^s^-1^]
1	11	8.80 ± 1.99	10	2.088 ± 0.357	0.237	0.46	0.72	0.839	1.376
2	11	9.39 ± 2.18	10	2.792 ± 0.291	0.297	0.49	0.71	1.026	1.886
3	10	7.82 ± 2.51	13	2.430 ± 0.220	0.310	0.47	0.52	1.402	1.735
4	12	7.06 ± 1.45	16	2.416 ± 0.364	0.342	0.28	0.55	0.997	2.321
5	10	7.93 ± 2.63	16	2.316 ± 0.209	0.292	0.50	0.58	0.822	1.132
6	13	8.35 ± 1.68	16	2.513 ± 0.217	0.301	0.58	0.67	0.701	1.013
7	8	8.35 ± 1.68	16	2.787 ± 0.244	0.334	0.61	0.74	0.414	0.572
8	12	9.76 ± 2.27	16	3.533 ± 0.221	0.362	0.49	0.65	1.023	1.271
9	9	9.51 ± 2.28	19	3.053 ± 0.434	0.321	0.40	0.64	0.852	1.218
10	8	6.90 ± 1.57	19	3.549 ± 0.779	0.514	0.53	0.73	0.525	1.487

*M*O_2_ (mgO_2_ fish^-1^h^-1^) of *S*. *sprattus* with different mean wet mass (M_W_, g) at different water temperatures (T,°C) and related observations of minimum (Min) and maximum (Max) values for of swimming speed per body length (U, BL s^-1^) and the number of direction changes >90° (*M*, fish^-1^s^-1^), respectively.

### Image acquisition

Fish were filmed under constant infrared light conditions using a CCD-Camera (Ecoline TV7002, Security Center) above the respirometer and an illumination box covered with a light reflecting foil and infra-red LEDs below the respirometer-chamber (Fig 1). This design provided a bright and constant background against which the fish appeared as dark objects in the images. The grey value images (Fig 1) had a size of 320x240 pixel and were recorded with 4 Hz using the program i-Corder^®^ (V2T VISION TO TECHNOLOGY GmbH, Germany).

### Automated fish tracking

We developed a new fish tracking system based on available algorithms and concepts for multiple object tracking [29], but adjusted for the present tank design, fish size, group size, experimental design, visibility of fish in the respirometer chamber and the actual frame rate. Available software to detect animal movements was not directly applicable to our experimental design and tank size. For example, the program of Pinkiewicz [30] allows the tracking of a fish school (larger fish swimming mainly in the same direction) but is not applicable to our experimental design and the small species of fish. As *S*. *sprattus* are extremely sensitive to scale loss and handling, it is not possible to add colour tags on *S*. *sprattus* which may allow differentiating individuals on images by automated programs like SwisTrack (2008, version 4)[31].

**Fig 3:**
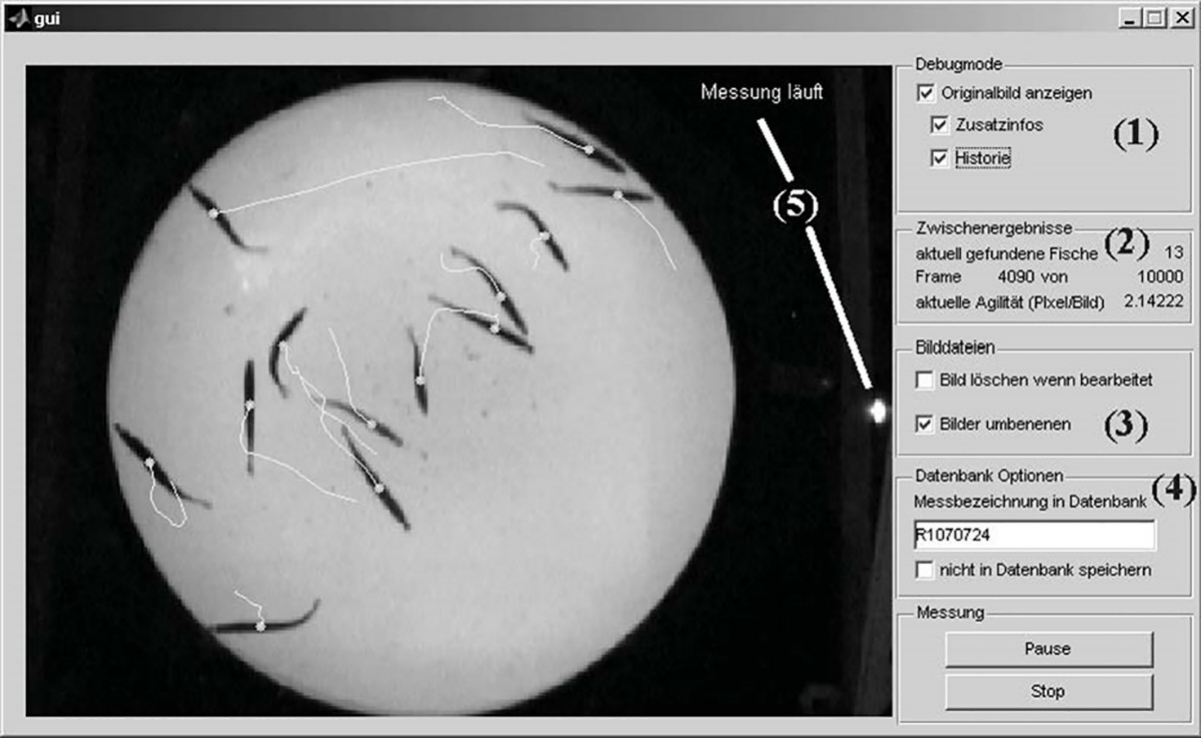
Graphical user interface (GUI). GUI of the fish-tracker for the automated tracking of sprat in an intermittent-flow respirometer. During the processing it was possible to show the original images with the option to display the centre points of each fish and the tracking history of the last twelve images as lines (1). Information on the actual tracked image (2) was given below. Image processing options (3) and database options (4) had to be adjusted before the start of the program. The IR-LED was powered only during measuring phases (5).

To analyse the movements of the fish, a program (hereafter: fish-tracker) running with MATLAB® (Version 7.3.0.267, The MathWorks Inc., Image-Processing-Toolbox Version 5.3, Database-Toolbox Version 3.2) was designed, which recognized fish movements as changes of fish positions from picture to picture and saved the x-y-positions of each fish in each frame in a database (MySQL). With a graphical user interface (gui) it was possible to visualize the fish centre locations on the actual image, along with the tracking history from the previous twelve frames (Fig 3). The processing of the fish-tracker is explained with the following steps:

1. Object identification and allocation: All Objects (fish and other particles) were identified by their grey values, which were higher than a predefined threshold value representing the background field. Fish were separated from other particles by a size threshold (number of pixels forming the object) and labelled with an identification-number (ID). The original grey value image was then converted into a binary picture, with identified fish being coded as one, whereas the background and non-fish pixels were coded as zero (subtraction method [29]). The geometric centre (centre of gravity) of the pixels representing a fish was calculated by the “first moment” method.
2. Estimation of expected fish positions: A Kalman-Filter [32] was used to estimate the expected positions of each fish on the following image. The Kalman filter is a method of combining noisy (and possibly missing) measurements and predictions of the state of an object to achieve an estimate of its true current state. This estimation filter was based on the detected positions of each fish in the last five to twelve images. The Kalman filter was extended by a nearest neighbour method in order to allow for multiple object tracking and to avoid any correspondence problems (also called “occlusion events”, [29]). This method determined the prospective position of all identified fish based on the individual positions of the previous image: For each object, the nearest neighbour (Euclidean distance) was identified regarding the calculated positions from the Kalman filter. The two objects from the current and the previous frame were assumed to be identical if there was a clear minimum distance and this distance was below a predefined threshold value. In situations where fish could not be clearly identified the following steps were undertaken: 1. If two fish were swimming directly upon each other, the ID for one of them was removed in this frame. In the following frame, the nearest object to the crossing location received a new ID. 2. If a fish was not clearly identified in the last five images its ID was removed from these five frames, but the same fish could be recognized in the subsequent frame as a new object with a new ID number. This made it not possible to track an individual over the complete experiment, but allows having enough simultaneous tracking events during each experimental interval.
3. Handling of overlapping objects: If objects were very close to each other, correspondence might be incorrect and cause the nearest neighbour method to fail. Therefore overlapping objects needed to be separated by another procedure: When two or more fish were overlapping, so that the surface area of this object exceeded the maximum area of an individual fish, these objects were separated by morphological erosion and dilation [33,34]. When two fish were swimming directly one after another they formed an object, which exceeded the maximum length of an individual fish. These two fish were then separated along the axis of the second moment. This means that the positions and directions of the two fish were determined by a segmentation of the oversized object along the major axis of an ellipse, equivalent to the objects, and under consideration of their orientation and mass centre (see “regionprops” in MATLAB®).

### Determination of swimming speeds and direction changes

To translate the position data obtained from the fish-tracker to absolute swimming speeds of the fish, the velocity of flow in the respirometer chamber had to be considered. The velocity pattern was analysed with a small neutrally buoyant plastic ball (drifter) which was floating freely in the water column. Its drift path was monitored with a frequency of 4 Hz and positions were analysed by the fish-tracker. Drift-experiments without fish were undertaken at six different flow-through rates ranging from 1.62 to 4.50 L min^-1^. There were no indications of turbulences induced by the current based on the observations of swimming fish and the drifter experiments, nor were fish showing any behaviour indicating stability problems.

A second program was designed (in MATLAB^®^), which analysed the position-data of the drift-experiments to develop a velocity model. The mean water velocity was calculated from all obtained x- and y-positions of the drifter and the respective time steps. The estimated velocity field had an elliptic form due to the unidirectional inflow of water in the chamber and the flow rate increased from the middle of the tank within the ellipse and decreased at the edge of the chamber. Mean calculated drift speeds ranged from 0.16 to 4.97 cm s^-1^, depending on the position in the tank. The model was used to estimate the water velocity at each x-y-position at a given mean flow rate through the chamber. The resulting value was then added to the swimming speed of a fish at the same position, calculated from the x-y-positions from two consecutive images.

The swimming direction of a fish was determined from the difference of two positions between two successive images. The change in swimming direction of each individual fish was then calculated as the difference of swimming directions between two successive images. Mean swimming speeds (BL s^-1^; cm s^-1^) and direction changes (°fish ^-1^between two images) were calculated for each experimental interval from the x-y-positions of the fish and the derived estimates of their swimming directions. During a sharp turn (90–180°) a fish turned roughly in the same place, so that the covered distance during a sharp turn was negligible. Swimming speeds and turning rates were averaged per fish group and measuring interval (23 min) in order to relate swimming activities to mean oxygen uptake rates (*M*O_2_) of the respective measuring interval.

### Calculation of oxygen uptake rates

Correcting for chamber volume and the displaced volume of the fish, the actual oxygen uptake rate in one measuring interval in mgO_2_ h^-1^ of the fish group was calculated by linear regression:

$$V_{O_{2}}=C_{t}(V_{c}-V_{f})$$ (1)

*V*_O2_ = oxygen uptake (mgO_2_ h^-1^); C_t_ = mean decrease of the oxygen concentration in the respirometer (mgO_2_ L^-1^ h^-1^); V_c_ = chamber volume (ml); V_f_ = fish volume (ml). The total oxygen uptake was then divided by numbers of fish and fish wet mass after values for bacterial respiration were subtracted [27].

### Data selection

The first 1–3 hours of experiments were excluded from further analysis (see Fig 2) because oxygen uptake of the fish group was possibly affected by elevated levels of stress hormones. Many fish were swimming in close proximity to the tank walls during these intervals. This stress-related swimming behaviour was not adequately represented by mean swimming speeds or turning manoeuvres. During our experiments, most individuals calmed down and showed normal swimming behaviour within three hours, which was controlled by inspection of the respective images.

Measurements of standard or routine metabolic rates of pelagic fishes are often conducted in intermittent-flow respirometers [25] with preferably short measuring intervals. Initial acclimation phases are then excluded as these are largely influenced by higher and more variable spontaneous swimming activities [27,28,35]. The estimation of the relation between variable spontaneous swimming patterns and metabolic costs actually requires such contrasting levels of swimming activities [13,22]. Thus, the idea of the present study was to utilize the measurements from acclimation phases, without the initial period of recovery from handling stress.

### Model selection

Based on the current understanding of metabolic costs for fish and published information on the mass- and temperature dependency of metabolic rates, a set of biologically reasonable models was formulated and subsequently fitted to the data. Each candidate model represents a biological hypothesis.As the present data are not appropriate for estimating mass-and temperature dependency of a standard metabolic rate (SMR), we adopted the SMR term in our model formulations (Table 2) from [27] where a more comprehensive data set was available encompassing a larger temperature- and body mass range. In these previous experiments, fish always showed slight spontaneous movements and were never totally quiescent. The data from our new experiments allowed quantifying the additional costs referring to the difference between increased, variable swimming movements and slower, more constant movements during SMR. This prompted us to subtract the lowest observed activity values from the current experiments (swimming speeds of 0.28 BL s^-1^ and turns>90° of 0.41 fish^-1^s^-1^) as a constant from each actual swimming speed (and turning rates) in some of the tested model formulations (Table 2). These constants are assumed to represent the minimum activity already included in the previously measured SMR by Meskendahl [27].

**Table 2:** Predictive models for metabolic rates.

No	Formula	K	Estimate	*RSS*	AIC	AICc	Δ _*i*_
M1	*M*O_2_ ~ a M_W_^1.073^ e^(0.078 T)^ + b (U- 0.28)^v^ e^(c1 T)^ + d (M—0.41)	a	0.036	27.1	175	-193	2.74
		b	1.590				
		v	1.791				
		c1	0.104				
		d	1.002				
M2	*M*O_2_ ~ a M_W_^1.073^ e^(0.078 T)^ + b (U- 0.28)^v^ e^(0.078 T)^ + d (M—0.41)	a	0.032	27.6	177	-193	2.69
		b	1.880				
		v	1.422				
		d	0.910				
M3	*M*O_2_ ~ a M_W_^1.073^ e^(0.078 T)^ + b (U- 0.28)^v^ + d (M—0.41)	a	0.049	39.2	255	-159	36.44
		b	12.01				
		v	2.458				
		d	0.795				
M4	*M*O_2_ ~ a M_W_^1.073^ e^(0.078 T)^ + b U^v^ e^(c1 T)^ + d M	a	0.025	26.3	168	-195	0.00
		b	0.704				
		v	2.999				
		c1	0.124				
		d	0.888				
M5	*M*O_2_ ~ a M_W_^1.073^ e^(0.078 T)^ + b U^v^ e^(0.078 T)^ + d M	a	0.018	27.7	178	-192	3.04
		b	1.233				
		v	1.934				
		d	0.716				
M6	*M*O_2_ ~ a M_W_^1.073^ e^(0.078 T)^ + b U^v^ + d M	a	0.039	41.6	268	-153	42.21
		b	4.34				
		v	3.135				
		d	0.506				
M7	*M*O_2_ ~ a M_W_^1.073^ e^(0.078 T)^ + b (U- 0.28)^v^ e^(c1 T)^	a	-0.001	42.6	274	-151	44.42
		b	2.015				
		v	0.321				
		c1	0.047				
M8	*M*O_2_ ~ a M_W_^1.073^ e^(0.078 T)^ + b (U- 0.28)^v^ e^(0.078 T)^	a	0.005	54.8	327	-129	66.54
		b	1.209				
		v	0.375				
M9	*M*O_2_ ~ a M_W_^1.073^ e^(0.078 T)^ + b U^v^ e^(c1 T)^	a	-0.010	39.7	258	-158	37.59
		b	2.015				
		v	0.604				
		c1	0.050				
M10	*M*O_2_ ~ a M_W_^1.073^ e^(0.078 T)^ + b U^v^ e^(0.078 T)^	a	-0.004	52.4	317	-133	62.23
		b	1.240				
		v	0.696				

*M*O_2_ (mg O_2_ fish^-1^ h^-1^) of *S*. *sprattus* using wet mass (M_W_, g), temperature (T,°C); mean swimming speed (U, BL s^-1^) and the number of turns >90° (M, fish^-1^s^-1^) as explicative variables. *n* = sample size, K = parameter, AIC = Akaike information criterion, AICc = n*log(RSS/n)+2K+(2K(K+1))/(n-K-1), **Δ**_*i*_ = difference between the AIC of the best fitted model and that of model *i*

We modelled the relationship between swimming speed (*U*) and oxygen uptake as a power function [36–38], which is based on hydrodynamic principles and allows for species comparisons when SMR is different [39,40]. The data of the present study was not sufficient to estimate any body mass effects on swimming speeds as they were obtained from fish with only slightly different body masses (Table 1). We therefore used swimming speeds in body length per second (BL s^-1^) to relate the fish’s activity to metabolic rate instead of cm s^-1^, because fish swimming speed varies with fish body mass [41] and therefore with total fish length. The energy available for moving is always related to the muscle mass, which itself is a function of size [42]. This means that a small fish spends more energy in relation to its body size than a larger fish for swimming with the same speed in cm s^-1^. In addition to mean swimming speeds, turning rates [18] or turns with high angles [12] may account for a substantial part of the metabolic costs during spontaneous activity [18]. We therefore calculated the turning rates (°s^-1^) for each tracked fish per experimental interval. Mean turning rates per interval were in the range between 87 and 194°s^-1^ (mean = 135% ± 20) and were not directly correlated (linear regression, r^2^ >0.2) with the observed oxygen uptake rates or mean swimming speeds, respectively. The observed turning rates were therefore divided into three categories: 1) turns with less than 90°s^-1^, 2) turns between 90 and 180°s^-1^ and 3) turns larger than 180°s^-1^. Overall, turning rates with small turning angles (<90°) were observed much less frequently than sharper turning movements. The proportion of turns <90° per experimental interval was between 4–39% (mean = 17%). Turning rates with higher angles (90–180°s^-1^) accounted for 27–79% (mean = 64% ± 8) of all turning movements and turns with more than 180°s^-1^ accounted for 1–52% (mean = 17% ± 9) of total turning movements. Thus, most turns were conducted with angles higher than 90°s^-1^. As turning with small angles (<90°s^-1^) were performed less frequently and were to some extent included in mean swimming speeds, we tested the number of turns >180° (fish^-1^s^-1^) and number of turns >90° (fish^-1^s^-1^) for explaining variability in oxygen uptake in addition to mean swimming speeds in our modelling approach. Such movements with high turning angles are known to account for a significant part of spontaneous activity costs [12]. Simple linear regression for the single data sets (per experiment) resulted in a higher regression coefficient (r^2^) when oxygen uptake was modelled as a function of turns >90°, than with other estimates of manoeuvrings. Thus, we included only turns >90° in our modelling approach for the complete data set. Although accelerations of single fish could be determined from the data of tracked fish, mean values for the fish groups were always close to zero and were not correlated with mean *M*O_2_.According to findings by Webb [11] some rectilinear accelerations and decelerations are also represented by frequent sharp turns.

We used non-linear regression analysis (nonlinear least-squares, [43], package = “stats”) to relate spontaneous swimming patterns (turns >90° and mean swimming speeds) to oxygen uptake of *S*. *sprattus* and using the Akaike information criterion (AIC) for model selection and comparison [44–46]. The criteria used to identify the model with the highest probability of being the best were lower values for AIC, AICc and Δ*i* values as well as analysis of regression residuals. Further model validation included tests for variance homogeneity (residual plot) and normally distributed measurement errors (Shapiro-Wilk-test) following the recommendations by Ritz and Streibig [47]. Another subordinate criterion in model selection was the model performance when extrapolated to higher swimming speeds than observed here, which is an important aspect for later applications. We therefore inspected the results of model extrapolations to higher swimming speeds of up to 3 BL s^-1^, which is the overall swimming speed observed for *S*. *sprattus* during feeding on high prey concentrations [8] and is also the theoretical “optimal foraging speed” defined by Ware [41,48] who found that with increasing food concentration, fish increase their swimming speed up to a level of ~ 3 BL s^-1^. The optimal foraging speed is defined as speed that maximizes the fish growth over time in relation to the net food intake per unit time, the standard metabolic rate and the costs of swimming, respectively.

### Cost of Transport (COT)

The metabolic cost of transport (COT) is a common measure for the energy required for a fish to swim a given distance. It is typically calculated for fish swimming at *U*_*opt*,_ which is the straight-line swimming speed associated with minimum energetic costs per unit distance [35,36]. COT can also be determined for various observed swimming speeds (*U*) in order to define relationships between swimming costs and temperature. It provides a conservative measure for evaluating effects on changing water temperature on swimming energetics [49] and can be calculated from our final model divided by a given selected swimming speed (0.57 BL s^-1^) and setting turns to a constant low value (~ 0.41 turns fish^-1^h^-1^; the lowest value observed; Table 1). We calculated the *M*O_2_ for an 8 g fish with a length of 10 cm and converted the oxygen uptake from mgO_2_ fish^-1^h^-1^ into mgO_2_ g^-1^s^-1^. Swimming speeds were converted from BL s^-1^ into cm s^-1^ in order to finally calculate COT as mgO_2_ g^-1^cm^-1^.

## Results

### Swimming characteristics

**Fig 4:**
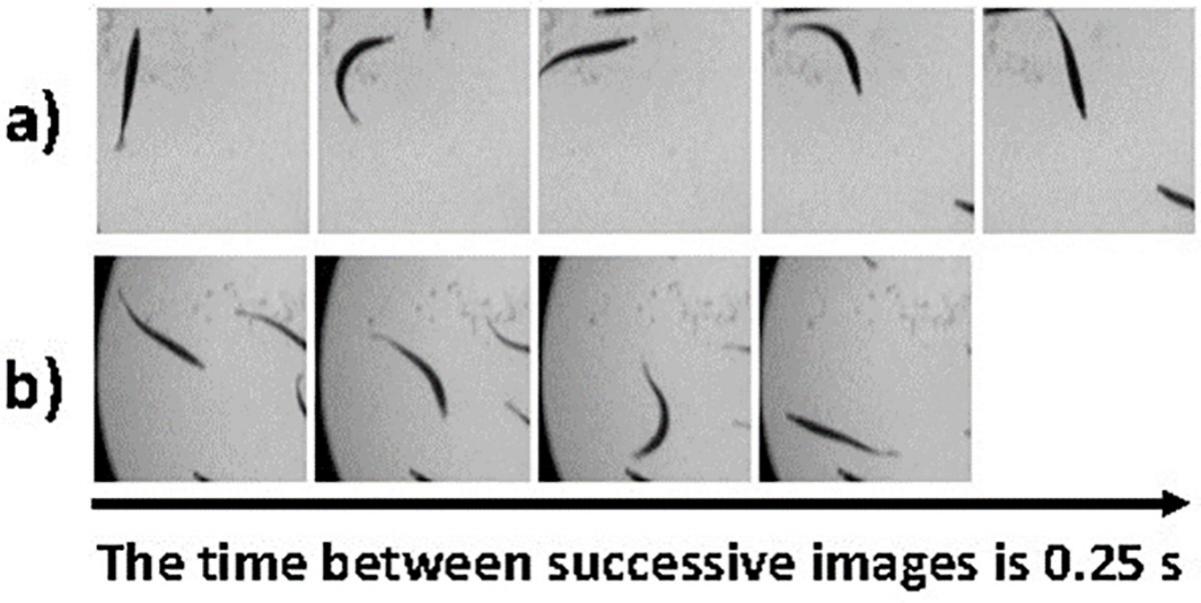
Example of typical turning movements of an individual sprat during a respirometry experiment. In the upper sequence (*a*) one fish shows two successive turns within 1.75 s. In the second sequence (*b*) one turn of ~180° was performed within less than one second.

Within each experiment, mean swimming speeds and the number of turns >90° per interval varied over time and resulted in variable oxygen uptake rates (Fig 2). Mean swimming speeds during experiments ranged from 0.28–0.74 BL s^-1^ (Table 1) and the coefficient of variation (CV; %) in swimming speed during the acclimation phase was similar for all temperatures ranging from 15.8% at 19°C to 19.0% at 10°C. Only for experiment No. 3 at 13°C was variability in swimming speed was much lower (2.7%). These results indicate that spontaneous swimming was generally variable during the acclimation phases of experiments (Fig 2), but not different among the different tested temperatures (Table 1). The mean number of turns >90° per interval was also variable between experiments with values ranging from 0.414 to 2.321 fish^-1^s^-1^ and the lowest CV of 6.8% in experiment No. 3 and highest CV of 20.8% in experiment No. 10. Thus, the number of turns >90° was also highly variable within experiments, but there was no trend with water temperature. An individual fish was able to perform one turning movement of ~90° with maximal bending of its body within 0.25 s or two turns within 0.75 s, but also turns of ~ 180° within one second (Fig 4). This is also reflected by the high mean number of turns >90° of up to 2.3 fish^-1^s^-1^ for one experimental interval (Table 1).

### Spontaneous swimming costs

Mean oxygen uptake per experiment ranged from 2.088 mgO_2_ fish^-1^ h^-1^ at 10°C to 3.549 mgO_2_ fish^-1^ h^-1^ at 19°C (Table 1). The coefficient of variation for *M*O_2_ was lowest for experiment No. 8 with 6.3% and highest for experiment No. 10 with 19.1%. For a dataset of 222 metabolic rate measurements and the corresponding swimming speeds and numbers of turns >90° different models (Table 2) were tested to describe the relationship between the metabolic rate (*M*O_2_, mgO_2_ fish^-1^h^-1^) of *S*. *sprattus* and mean swimming speed (U, BL s^-1^), number of turns (M, fish^-1^s^-1^), body mass (M_W_; g) and water temperature (T,°C). All tested models were based on the same data set from 9 experiments (experiment 5 was excluded during the modelling process).

**Fig 5:**
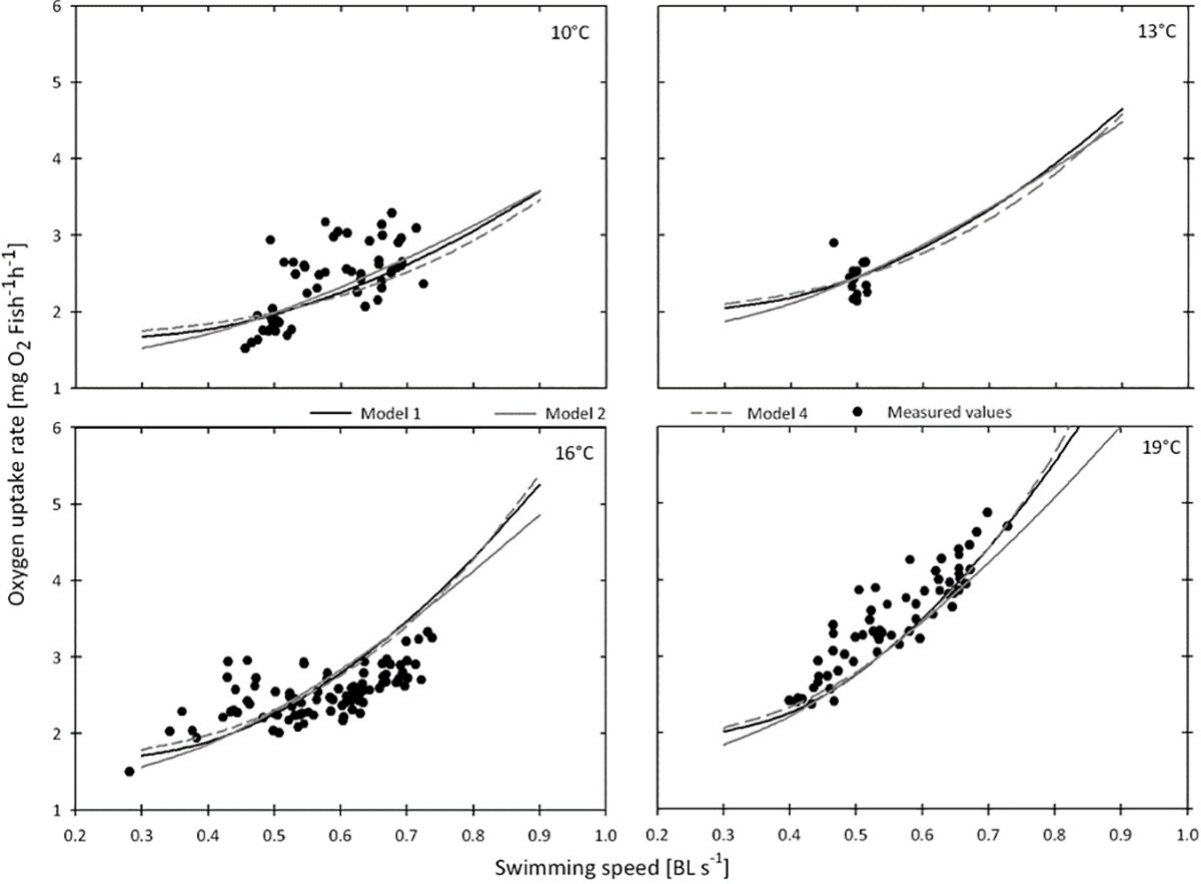
Oxygen uptake rates at different temperatures and swimming speeds. *M*O2; (mgO2 fish^-1^s^-1^) of sprat measured at temperatures between 10 and 19°C (black dots) over swimming speeds (BL s^-1^) and related model estimates (M1, M2 and M4; see Table 2) for the mean number of turns and mean wet weights observed at the respective temperatures (°C).

Models M7-M10 represent approaches where the number of turns >90° were not included in order to evaluate the necessity of incorporation of such manoeuvres.These models are unlikely to be good predictors of swimming costs in *S*. *sprattus* as they have high AIC values (Table 2) and negative estimates for the parameter *a* (representing standard metabolism). The Models M1-M3 represent approaches with subtraction of a constant from the actual swimming speeds and turning rates (see Material and Methods), whereas models M4-M6 are similar to M1-M3, but without the subtraction of a constant. Model M4 had the lowest AIC and AICc. Within the tested range of swimming speeds, model predictions of M1, M2 and M4 are very close together (Fig 5).

If calculated with M4 for a 7g fish at 16°C the costs for sharp turns at a frequency of 1 s^-1^ doubled the metabolic costs compared to those with zero turns but at a likewise a moderate swimming speed of 0.28 body length ^-1^. At the high rate of turns these account for 52% of total metabolic cost. With no turns the cost for swimming doubled if the swimming speed was doubled from 0.28 to 0.56 BLs^-1^ but increased by a factor of 4.5 if tripled to 0.84 BLs^-1^. At 0.84 BLs^-1^ swimming accounted for 81% of the total cost.

### Cost of transport (COT)

**Fig 6:**
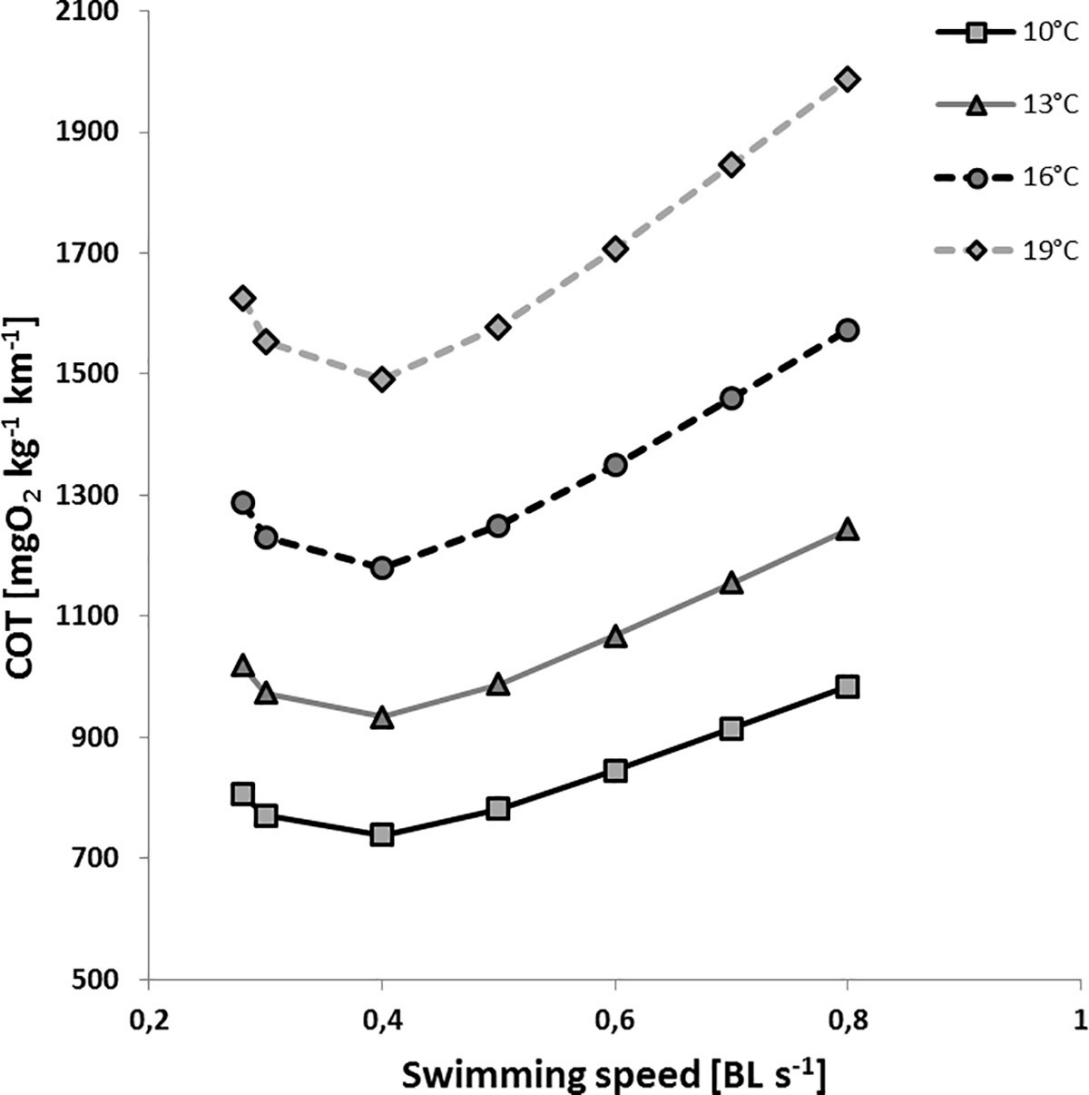
Cost of transport as a function of swimming speeds. **COT (mgO**_**2**_ **kg**^**-1**^**km**^**-1**^**)** at different swimming speeds (BL s^-1^) and temperatures of 10°C (squares), 13°C (triangles), 16°C (circles) and 19°C (diamonds) calculated by application of model M2 with a fish wet weight of 8 g and mean turns>90° of 0.41 (fish^-1^h^-1^). See Materials and Methods for further details.

The cost of transport (mgO2 g^-1^cm^-1^) calculated by the application of model M2 as a function of body lengths (BL s^-1^) revealed that the minimum values (COT_min_) were always obtained at a swimming speed of 0.4 BL s^-1^ (Fig 6). Thus, this swimming speed can be termed the optimum swimming speed (*U*_*opt*_) under the given assumption that turns are low and constant. The obtained COT_min_ values were clearly lower for low than for high ambient temperatures and reflect the general temperature dependency of swimming costs (see Fig 6). If calculated with M2, where both temperature exponents were fixed to the estimate of standard metabolism of 0.078, corresponding to a Q_10_ of 2.1 [27] the Q_10_ of COT was likewise 2.1. In contrast M4 included a second temperature exponent for the swimming term, which was estimated from the data as 0.124 corresponding to a Q10 of 3.45. Hence, on the basis of M4 the overall Q10 increases with increasing swimming speed from 2.1 towards 3.45.

## Discussion

### Fish behaviour and tank effects

The present results indicate that the higher oxygen uptake rates in the beginning of the experiments are mainly explained by higher swimming speeds and more turns >90° (Fig 2) and were hardly affected by handling stress. The exclusion of the first 1–3 hours has most likely removed elevated oxygen uptake rates which are not directly reflected by higher swimming activities. Handling stress in fishes can generally result in catecholamine stimulation and the subsequent release of cortisol [50]. This can increase cardiac output, oxygen uptake and the mobilization of energy substrates, but it is still unclear how long elevated levels of cortisol or other stress hormones are sustained [50] and to what extent this is not reflected in increased activity. Davis & Schreck [51] discovered that elevation in oxygen uptake rates in juvenile coho salmon was largely eliminated within 1 h after handling stress. As fish in our experiments were pre-acclimated to the smaller tank size and experimental temperatures, stress was mainly induced by the careful transfer into the respirometer. Thus, we assumed that fish were no longer stressed when they showed their normal swimming behaviour which was evaluated by visual inspection.The continued elevated activity and MO2 may reflect an incomplete habituation to the new chamber.

The observed low swimming speeds (0.28–0.74 BL s^-1^) and high numbers of turns (0.414–2.321 fish^-1^s^-1^) in the respirometer were probably influenced by the artificial confinement of the fish. The tank was relatively small due to restrictions imposed by respirometry techniques [25] and this can affect the swimming characteristics of fish [10,13]. *S*. *sprattus* is a schooling fish and individuals benefit from swimming in groups as this reduces costs of locomotion [52].*Sprattus sprattus* naturally shows variable spontaneous swimming movements as they are not always swimming in polarized schools, but also swim in random patterns under low light conditions during the night [53] or during feeding where *S*. *sprattus* and *C*. *harengus* show frequent sharp turns [7,8]. Thus, energetic costs for spontaneous swimming are of great interest for bioenergetic models and such behaviour was represented in our experiments.

### Conclusions from model selection

We formulated and tested ten models to relate *M*O_2_ of *S*. *sprattus* to swimming patterns (speeds and turns >90°) and water temperature (Table 2). In theory, two different approaches were applied: in some model formulations, a constant for swimming activity (turns and speeds) related to standard metabolism was subtracted from the turns and speeds observed (Models M1-M3 and M7, M8), whereas the other models (M4-M6, M9 and M10) had no constant (Table 2). From these models, M1 and M2 had the same AICc value of -193 and M4 had the lowest AICc value of -195. As recommended by Burnham and Anderson [44], AICc and Δ*i* values should be used as criterions for model selection from a set of reasonable models. As a rough guideline, Δ*i* values around 2 mean that a model is considerably supported, whereas models with Δ*i* values between 4 and 7 are less supported and models with Δ*i* >10 have no support. Hence, we needed to consider the models M1, M2 and M4 as reasonable approximations for our data set. It was therefore necessary to further decide which one should be chosen as the final (“best fit”) model. None of the models can explain all the variability in the measurements (Fig 5), which might partly be an effect of the long measuring intervals. With a higher data resolution, one could possibly get better fits between observed swimming behaviour and oxygen uptake.As discussed above, the measuring intervals were as short as possible in a set up were sufficient space for natural swimming patters of a schooling fish represented a strong restriction. The model predictions for the three models M1, M2 and M4 are actually very close together in the range covered by observations (Fig 5). Only when extrapolated to higher swimming speeds, the differences between the models become obvious. For instance, when applied to an 8 g fish at 16°C with a turning rate of 1 turn >90° fish^-1^s^-1^ M1 predicts 52.2 mgO_2_ fish^-1^-h^-1^ at 3 BL s^-1^ while M2 and M 4 predict 28.7 and 139.8 mgO_2_ fish^-1^-h^-1^, respectively. M1 includes a separate term for the temperature dependency of swimming costs and has a slightly lower AIC value than M2, but the corrected AICc was similar for both M1 and M2. In M2, the body mass exponent of *k* = 1.073 and the temperature exponent of *c* = 0.078 of standard metabolism were fixed to the values obtained by Meskendahl *et al*. [27]. Although M4 has the lowest AICc value and should therefore be the preferred model, this model results in extraordinarily high metabolic rates when extrapolated to swimming speeds beyond the observed range. Thus, M2 is recommended as final model when applied to higher swimming speeds (>0.8 BL s^-1^), but for applications within the swimming speed range of 0.2–0.8 BL s^-1^ model M4 reflects the interaction between temperature and swimming costs better than the other model formulations.

### Temperature effects

In the present study *S*. *sprattus* showed no differences in swimming speeds and turning rates with temperatures (10 to 19°C). Positive correlations between speeds and temperature are assumed to occur only below certain water temperatures [54–56]. Thus, in bioenergetic models for alewife, *Alosa pseudoharengus* [57], and *C*. *harengus* [58], swimming speed was modelled as a function of temperature only below 9°C and swimming speeds were set to be constant at higher temperatures.In our study total metabolic costs showed a greater increase with increasing temperature at the swimming speeds observed (Fig 5). Thus, metabolic costs were best described with an additional exponential function of temperature within the swimming speed term (Table 2). We inspected the swimming speed term (*b* * U ^*v*^) separately in order to evaluate the changes in swimming costs with temperature, especially as there is a sharp increase in swimming costs from 16°C to 19°C (Fig 5). We therefore fixed all other parameters in the model formulation to the values presented in Table 2 and estimated the parameter *b* separately for each temperature subset. This analysis revealed that *b* was similar below 16°C, but much higher at 19°C. This explains why M4 being a model with a separate and high temperature exponent for swimming costs (with *c* = 0.124; see Table 2) has the lowest AIC and can predict the steepness of increase in oxygen uptake at 19°C better than other model formulations.

The energy expenditure calculated per unit distance (COT) quantifies the foraging or cruising efficiency [35]. Costs of transport calculated here as a function of swimming speed followed the J-shaped form described in previous studies [59,60]. As swimming speed increases, the initial decline of COT is due to a decrease in the relative contribution of SMR to the overall metabolic demand. At the optimal swimming speed (*U*_opt_), COT reaches a minimum and thereafter increases as *M*O_2_ rises with swimming speed [36,59,60]. In the present study COT is increasing at higher speeds (up to 0.8 BLs^-1^) above the values obtained at the lowest speed (see Fig 6). Examples from other species suggest that swimming costs might be influenced by water temperature [61]. Hein & Keirsted [49] found that COT_min_, calculated for a range of straight-line swimming speeds of 22 different fish species, increases with increasing temperatures amongst as well as within species. Depending on the model formulation the temperature effect on COT is either described with a Q10 of 2.1 (taken from [27] and fixed] or with a Q10 which increases with swimming speed from 2.1 to above 3.0. This is clearly higher than the value described by Hein & Keirsted [49] with Q_10_ = 1.3 for a combined analysis of various fishes. The difference may be related with much more variable swimming patterns of spontaneous movements compared to straight-line swimming. Low and variable swimming speeds might be less energy efficient than constant straight-line swimming and are probably more influenced by water temperature.

### Classification of spontaneous active metabolic rates

The spontaneous activity metabolism of *S*. *sprattus* at the average activity observed in our experiments with a swimming speed of 0.57 BL s^-1^ and a mean number of turns >90° of 1.096 fish^-1^ s^-1^ is only 1.3 (at 9°C) to 1.1-times (at 19°C) the costs associated with standard metabolism as estimated by Meskendahl *et al*. [27]. It should be noted, however, that swimming speeds were not registered in [27] and thus the SMR is not comparable to the first additive term in our models formulations. SMR in [27] represents the sum of our first term and contributions from the two activity terms with unknown values for swimming speed and rate of sharp turns. This amount of increase of MO_2_ in relation to standard metabolism is in line with measurements by Sirois & Boisclair [62] for the “spontaneous activity metabolism” of Brook Trout (*Salvelinus fontinalis*), which was 1.0 to 1.4-times the costs associated with standard metabolism. Thus, in some cases there was no or almost no difference between SMR and an elevated activity associated with spontaneous swimming movements. This is basically caused by the spontaneous activity already included in SMR measurements of such pelagic fishes where complete inactivity is not a natural state. Most measurements of *M*O_2_ in fishes to detect SMR have not concurrently measured activity [24] but there are also some important exceptions [6,10,18,22,62,63,64]. The problem with pelagic schooling fish and their variable degree of activity is that the metabolic level cannot be as easily categorized as into either standard or routine but there is rather a continuum from a theoretical minimum to ever higher rates at ever more activity. A theoretical minimum could be derived from the first term of our model formulations of M4, representing metabolic cost at swimming speed zero without sharp turns. Sirois & Boisclair [62] introduced the term “spontaneous activity metabolism” and several investigations focused on metabolic costs of spontaneously active fish [6,10,18,22], but there is still no generally accepted term for this metabolic level.Spontaneous swimming in fish was defined as a swimming mode involving both steady and unsteady components [9,22], whereas the latter includes turning manoeuvres, accelerations and decelerations [9,65]. Tudorache [22] found relatively low swimming speeds and high degrees of manoeuvring during spontaneous swimming of surfperch, similar to the results of the present study. The metabolism of *S*. *sprattus* from the present study reflects mainly an elevated routine metabolic rate under increased spontaneous activity (with sharp turning movements) and could be termed “spontaneous activity metabolism”. This metabolic rate should be clearly distinguished from the previously measured metabolic rates during the routine phase and under less variable swimming activities [27].

### Comparison of metabolic rates related to mean swimming speeds

The best fit model M2 describing the relationship between *M*O_2_ and swimming characteristics resulted in a swimming speed exponent of *v* = 1.422. Thereby *v* represents the steepness of the increase of *M*O_2_ with increasing activity [66] and can be directly used for comparisons among species [39]. This exponent is in the range of swimming speed exponents in power functions described for straight-line swimming fishes, ranging from 1.1–3.0 [36]. In most studies on clupeoids, exponential functions were used to describe the relationship between swimming speed and metabolism [67–69]. Exponential functions relating *M*O_2_ to swimming speed are basically useful for the extrapolation of metabolism to zero swimming speed in order to get estimates on standard metabolism [40], but are inadequate for direct comparisons among species when standard metabolic rates are different [39]. This makes direct comparisons of swimming costs among clupeoid fishes difficult. We therefore determined the increase in oxygen uptake resulting from a doubling in swimming speed within the measured range of the respective study (Table 3) in order to allow for a solid comparison of our results with other studies on related species. Thereby, *M*O_2_ of *S*. *sprattus* increased 2.62-fold with an increase in swimming speed from 0.4–0.8 BL s^-1^, which is slightly higher than factors found for other clupeoids derived from experiments at various speeds, ranging from 1.70-fold elevation in MO_2_ for *Engraulis mordax* [67] to 1.96-fold for the pilchard *Sardinops sagax* [66]. The observed mean swimming speed of *S*. *sprattus* with 0.57 BL s^-1^ was somewhat lower than the mean routine swimming speed reported for other clupeoids of 1.32 BL s^-1^ (Table 3), but relatively close to that of *S*. *sagax* [68], where also similar metabolic rates were measured. To the best of our knowledge, there are no comparable measurements of metabolic costs for high turning rates or direction changes like those from the present investigation for related species.

**Table 3:** Increase in metabolic rate (MR) with a doubling in swimming speed (*U*) in different studies.

		Swimming	T	M_W_	TL	MR at *U*	Mean *U*	Range	
Species	Reference	mode	[°C]	[g]	[cm]	[mgO_2_g^-1^h^-1^]	[BL s^-1^]	of *U*	Factor
*S*. *sprattus*	present study	spontaneous	16	8.5	10.6	0.220	0.57	0.4–0.8	2.67
*Sardinops sagax*	Van der Lingen 1995	voluntary; constant	16	146	25.6	0.178	0.78	0.5–1.0	1.96
*Engraulis encrasicolus*	James and Probyn 1989	voluntary	16	6.3	8.8	0.087	1.82	1.0–2.0	1.63
*Brevoortia tyrannus*	Macy et al. 1995	forced; constant	20	283	25.5^x)^	0.197	1.62	1.0–2.0	1.65
*Engraulis mordax*	Boggs 1991	forced; constant	17	8.7	8.1	0.264	1.07	1.0–2.0	1.70

T = temperature; M_W_ = wet body mass; TL = total length

^x)^ = fork length.

### Model application to feeding behaviour

We observed that biting of *S*. *sprattus* events during feeding on nauplii of *Artemia salina* or *Acartia tonsa* adults occur primarily during upward swimming [7,8]. At the upper end of the prey patch *S*. *sprattus* display a sharp turn (>90°), followed by a downward swimming burst with mean speeds around 2–3 BL s^-1^ [8]. Thus, the metabolic costs of turns >90° are of particular interest to assess the costs for feeding in *S*. *sprattus*. These feeding experiments were, however, conducted with smaller fish (~6 cm) in a larger tank than in the current study. This resulted in slightly different swimming behaviour when no food was supplied or prey concentration was very low. *S*. *sprattus* showed lower numbers of turns (~0.22 turns >90° Ind^-1^s^-1^) than the lowest values obtained in the current investigation where fish turned very often due to the small chamber size imposed by respirometry technique. Consequently, the current model M2 needs to be modified when applied to swimming speeds and turns >90° of *S*. *sprattus* during feeding on copepods. Here the last term, where a constant for the lowest observed turns was subtracted, needs to be modified. Using for example a value of 0.22 (as observed in the feeding experiments) instead of 0.41 results in parameter estimates of *a* = 0.0279, *b* = 1.679, *v* = 1.287 and *d* = 0.822 with an AIC of 179. This AIC is only 2 points higher than with the application of 0.41 in the previous model formulation (Table 2). So, both models are sufficient to describe the spontaneous metabolic costs in *S*. *sprattus*, but the latter version with the lower turn constant of 0.22 can be applied to the feeding behaviour as observed in the laboratory. Based on these results and new data on feeding activities of *S*. *sprattus* in the field (not available yet), it will be possible to relate mass gains and energy losses determined by activity patterns associated with feeding. This will further enhance our understanding of the predation impact by *S*. *sprattus* in the Baltic Sea and allow to model growth potential of *S*. *sprattus* under different ecological regimes in the Baltic Sea ecosystem.

## Supporting information

S1 File Basic data on metabolic rates, fish weight, temperature, number of turns and swimming speeds. (XLSX)
